# Combining an internal tension relieving technique with anterior cruciate ligament reconstruction (ACLR) reduces graft failure rate and improves functional outcomes: a systematic review and meta-analysis

**DOI:** 10.1186/s13018-025-05451-6

**Published:** 2025-01-07

**Authors:** Yixin Wen, Wei Huang, Minghui Li, Yong Jiang, Yibo Tong, Hongjun Mei, Junfeng Tan

**Affiliations:** 1 Department of Orthopaedic Surgery, The Fifth Hospital of Wuhan, Wuhan, Hubei 430000 China; 2 Department of Gynecologic and Oncology, Tongji Medical College, Hubei Cancer Hospital, Huazhong University of Science and Technology, Wuhan, Hubei 430000 China

**Keywords:** Anterior cruciate ligament reconstruction, Internal tension relieving technique, Graft failure, Knee score, Meta-analysis

## Abstract

**Purpose:**

Graft rupture is a significant cause of graft failure in anterior cruciate ligament reconstruction (ACLR). To address this issue, clinicians have combined the internal tension relieving technique (ITRT) with ACLR to improve graft stiffness, aiming to reduce the risk of graft failure. The purpose of this study is to compare the graft failure rates and clinical functional outcomes between ITRT-assisted ACLR and conventional ACLR.

**Methods:**

Following the PRISMA (Preferred Reporting Items for Systematic Reviews and Meta-Analyses) guidelines, a search was conducted in databases including Ovid, PubMed, Web of Science, Embase, Cochrane Library, Wanfang Data, CNKI, and VIP Medical Database for clinical controlled trials comparing the ITRT combined with ACLR to conventional ACLR. The search period spanned from the establishment of the databases to September 2024. Studies meeting the inclusion and exclusion criteria were selected, with two independent reviewers conducting literature screening, quality assessment, and data extraction. Data analysis was performed using RevMan 5.4 software. The evaluated outcomes included graft failure rate, Lysholm Knee Scoring Scale, Tegner activity score, Knee injury and Osteoarthritis Outcome Score (KOOS), International Knee Documentation Committee (IKDC) score, Visual Analog Scale (VAS) score, Single Assessment Numeric Evaluation (SANE), return to sport (RTS) rate, and knee joint laxity.

**Results:**

A total of 11 studies were included in the final analysis, with 1,339 patients (592 patients with ITRT-assisted ACLR and 747 patients with conventional ACLR). The combined analysis results indicated that, compared to conventional ACLR, ITRT-assisted ACLR showed significant advantages in reducing graft failure rates (RR = 0.44; 95% CI: 0.23, 0.83; *P* = 0.01), increasing return-to-sport rates (MD = 1.75; 95% CI: 1.05, 2.91; *P* = 0.03), and improving knee scores (including KOOS score and Tegner activity score) (all *P* values < 0.05). However, no significant differences were observed between the two approaches in terms of Lysholm knee score, VAS score, IKDC score, and knee joint laxity.

**Conclusions:**

This meta-analysis highlighted the significance and superiority of combining ITRT with ACLR compared to conventional ACLR, particularly in reducing graft failure rate and improving knee function outcomes. The ITRT-assisted ACLR procedure may represent the optimal approach for minimizing graft failure. However, given the limitations of short-term follow-up and reliance on retrospective studies, more randomized controlled trials and longer follow-up periods are needed to further evaluate the long-term graft failure rates and functional outcomes.

## Introduction

With the continuous development of the economy and society, and the gradual popularization of the concept of “sports health”, the enthusiasm of people for participating in sports activities has been steadily increasing. Against this backdrop, the incidence of sports-related knee injuries has also been on the rise. Anterior cruciate ligament (ACL) injuries are among the most common knee injuries [1–3]. As one of the most critical structures for maintaining knee stability, the primarily function of ACL limits excessive anterior translation of the tibia and excessive rotation of the knee joint [4, 5]. ACL injuries often lead to anterior and rotational instability of the knee joint, significantly affecting knee stability and function [6, 7]. If left unaddressed, these injuries can further damage the meniscus and articular cartilage, accelerate knee joint degeneration, and increase the risk of early symptomatic knee osteoarthritis [8–10].

Currently, arthroscopic ACL reconstruction (ACLR) remains the primary treatment for ACL injuries due to its advantages of minimal invasiveness and rapid recovery [11]. The goal is to maximize knee joint stability and restore the knee function as much as possible [12, 13]. It has been reported that in the United States, the annual incidence of ACL injuries is approximately 1 in 3,500, with about 400,000 patients undergoing ACLR each year [14]. Despite significant advancements in ACLR techniques over time, many patients still experience graft failure or suboptimal recovery of motor function [15, 16], partly due to the additional stress placed on the graft. Specifically, the graft undergoes a “ligamentization” process after ACLR [17], during which its strength and elasticity do not gradually increase; instead, they initially decrease sharply before slowly improving. During this period, the minimum strength of graft may be as low as 20% of its initial value [18]. Therefore, if the graft is subjected to excessive load during this time, it may become elongated or even rupture [19]. On the other hand, the quality of tendon-bone healing is closely related to early postoperative rehabilitation and functional recovery. Effective tendon-bone healing requires that the graft remains relatively stable within the surrounding bone tunnel [20, 21]. If the knee joint is immobilized for a prolonged period to facilitate tendon-bone healing, complications such as joint stiffness and muscle atrophy may occur. Conversely, during active rehabilitation training, knee movement inevitably places stress on the graft, leading to micromotion that can adversely affect tendon-bone healing.

To address this challenge, some researchers have proposed adding a tension-relieving band to the graft to help distribute the load, thereby protecting the graft, especially during the early stages of ligamentization [22]. A biomechanical study supports this approach, demonstrating that compared to standard ACLR, ACLR combined with an internal tension relieving technique (ITRT) improved graft stiffness and failure load, while reducing anterior tibial translation [23]. In the past five years, the ITRT has been adopted by several sports medicine centers worldwide for use in ACLR. EA Mackenzie et al., through a scoping review, found that ITRT-assisted ACLR increased graft strength and reduced elongation [24]. A retrospective study indicated that ITRT-assisted ACLR significantly reduced the risk of revision for patients [25]. However, the graft failure rates and clinical efficacy of ITRT-assisted ACLR remain unclear, and there is a lack of comprehensive literature summarizing the clinical outcomes of ITRT combined with ACLR. Therefore, the purpose of this study is to conduct a comprehensive meta-analysis to compare the differences in graft failure rates, return-to-sport rates, and knee functional outcomes between ITRT-assisted ACLR and conventional ACLR, providing a reference for clinicians in determining the optimal intervention for patients with ACL tears.

## Materials and methods

### Information sources and search strategy

The study adheres to the PRISMA (Preferred Reporting Items for Systematic Reviews and Meta-Analyses) guidelines for the execution of a systematic review and meta-analysis [26]. The study was registered with the International Prospective Register of Systematic Reviews (PROSPERO) under the ID CRD42024605559 prior to initiating the database search and study selection process. Searches were conducted in the following databases: Ovid, PubMed, Web of Science, Embase, Cochrane Library, Wanfang Data, CNKI, and VIP Medical Database. The focus was on clinical controlled trials comparing the combination of ITRT with ACLR to conventional ACLR, with a search period extending from the inception of each database to September 2024. The search utilized the following medical keywords and terms: [(anterior cruciate ligament reconstruction OR ACLR) AND (suture augment OR augmentation OR reinforce OR internal brace OR suture tape OR fiber tape)]. All retrieved literature was screened for relevance based on titles and abstracts to exclude unrelated studies. Subsequently, the full texts of the remaining articles were reviewed to identify those meeting the inclusion and exclusion criteria. Additionally, the reference lists of all included studies were examined to identify any other potentially eligible research.

### Inclusion and exclusion criteria

Inclusion criteria:: 1) Comparative studies of the combination of ITRT with ACLR versus conventional ACLR for treating ACL tears; 2) Study types include retrospective studies, prospective studies, and randomized controlled trials; 3) Studies providing sufficient clinical outcomes and functional results for further pooled analysis.

Exclusion criteria: 1) Biomechanical studies, letters, reviews, basic science research, surgical techniques, case reports, conference abstracts, animal studies, or meta-analyses; 2) Studies lacking sufficient follow-up data; 3) Studies involving duplicate samples or reports from the same patient cohort; 4) Studies with fewer than 10 patients; 5) Non-English literature was excluded due to resource constraints.

### Data extraction

Two independent researchers thoroughly reviewed the full texts of the included studies and extracted data from the final selected studies. The extracted data included the following: author, country, publication year, study design, sample size, follow-up duration, gender distribution, mean age, type of tension-relieving technique, fixation method, and clinical outcome measures (e.g., Lysholm score, Tegner activity score, IKDC score, KOOS score, VAS score, Single Assessment Numeric Evaluation (SANE), return-to-sport rate (RTS), knee joint laxity, and graft failure rate). To minimize data discrepancies, a third researcher was responsible for cross-checking and processing the data.

### Quality assessment

The quality of randomized controlled trials (RCTs) was evaluated using the Modified Jadad Scale [27]. The evaluation criteria included the following dimensions: 1) Generation of random sequences; 2) Randomization concealment; 3) Blinding; 4) Withdrawals and dropouts. Higher scores indicated better quality studies, with scores ranging from 1 to 3 considered low quality, and scores from 4 to 7 considered high quality.

For non-randomized controlled studies, the Newcastle-Ottawa Scale (NOS) was used for quality assessment [28]. The NOS evaluates three dimensions: the quality of cohort selection, comparability of cohorts, and outcome assessment, with a maximum score of 9 points. The scale includes four evaluation items: 1) Selection of the case group and control group, with clear definitions; 2) Comparability between groups (control of confounding factors); 3) Clear exposure assessment and consistency of investigation methods; 4) Explanation of non-response (loss to follow-up or refusal). A score above 5 is generally considered indicative of higher-quality studies. Any discrepancies in quality assessment between the two evaluators were resolved through detailed discussions. If disagreements persisted, a third evaluator was involved to reach a consensus.

### Statistical analysis

Data processing and analysis were performed using Review Manager (Version 5.4). For continuous variables, the effect size was represented by the weighted mean difference (WMD) or standardized mean difference (SMD), while for dichotomous variables, effect sizes were calculated using relative risk (RR) or odds ratio (OR). The results of the analysis were presented in the form of forest plots. Heterogeneity tests were performed on the data, following the guidelines of the Cochrane Handbook. An *I*² value less than 50% indicated low heterogeneity, 50-75% indicated moderate heterogeneity, and *I*² ≥ 75% indicated high heterogeneity. Sensitivity analysis was used to explain the significant heterogeneity observed between studies. A fixed-effect model (FEM) was employed when there was no heterogeneity or only mild heterogeneity among study results. In contrast, a random-effect model (REM) was applied in cases of moderate or significant heterogeneity. A *P*-value of less than 0.05 was considered indicative of a statistically significant difference between data.

## Results

### Results of literature screening

**Fig. 1:**
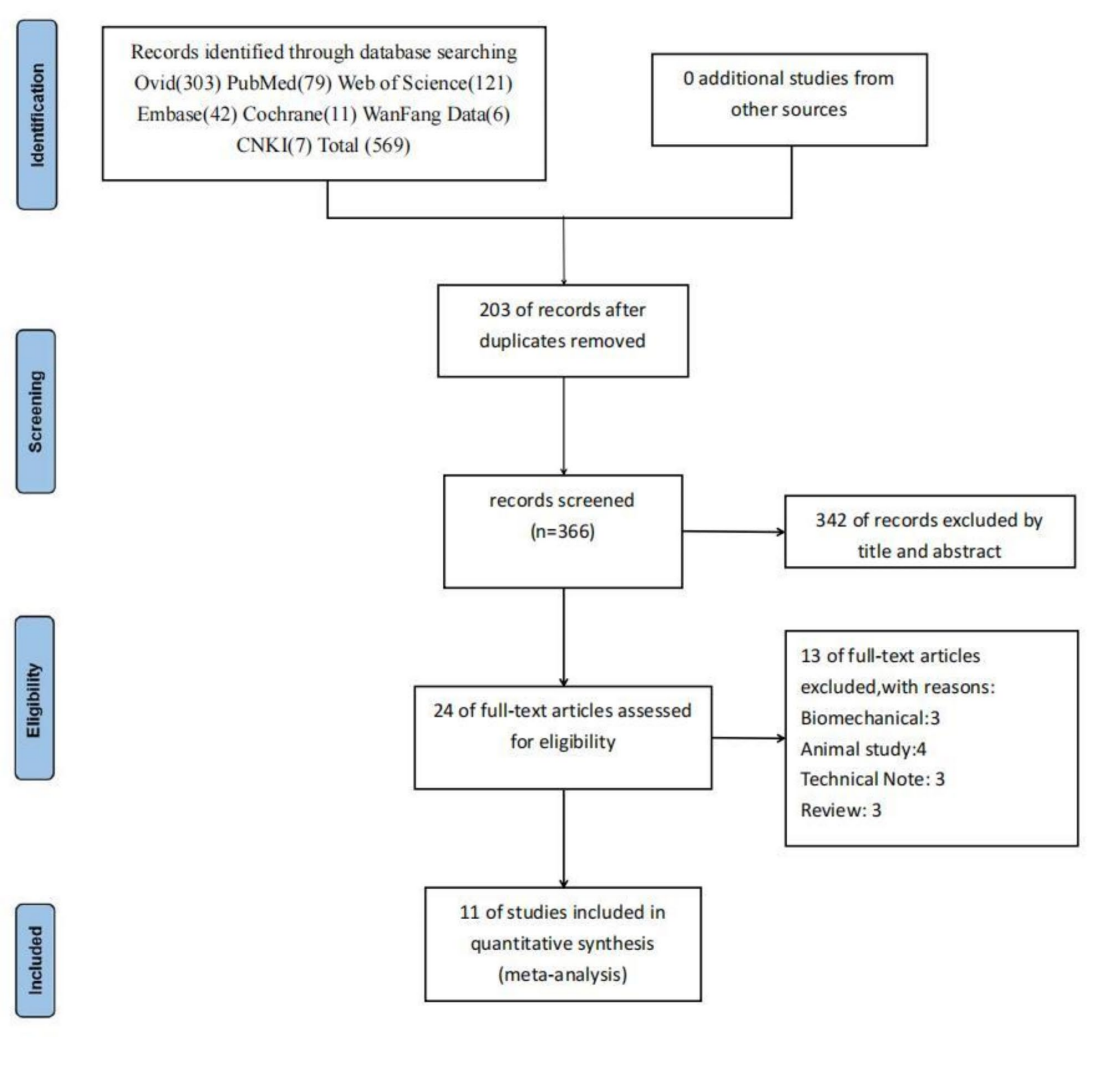
PRISMA flow diagram for included studies

The PRISMA flow diagram, shown in Fig. 1, illustrates the selection process. A total of 569 studies were initially identified through the database search. After removing 203 duplicates, 366 studies remained. The studies underwent an initial screening based on their titles and abstracts, which was then followed by a comprehensive full-text review in line with the established inclusion and exclusion criteria. As a result, 353 studies were excluded, and 11 controlled trials were ultimately included in the meta-analysis [19, 29–38].

This study included a total of 1,339 patients, with 592 patients receiving ITRT-assisted ACLR group ACLR and 747 patients undergoing conventional ACLR. The detailed characteristics of the included studies are presented in Table 1 and Table 2.

**Table 1:** Basic characteristics of all studies included

Study name	Region	Study design, level of evidence	Period	SEX, male /female, *n*	No.of patients, *n*		Age, year		Follow-up, mouth		QA
					SA	standard	SA	standard	SA	standard	
Allom 2022	AUS	Retro cohort ;3	May 2017 to June 2019	94/75	72	97	27.6 ± 10.0	25.6 ± 9.9	6		8^b^
Aujla 2021	AUS	Retro cohort ;3	April 2014 to December 2017	123/73	66	130	26.8 ± 9.5	27.5 ± 8.6	24		7^b^
Bodendorfer 2019	USA	RCS;3	September 2012 to March 2016	23/37	30	30	29.34 ± 7.55	29.65 ± 5.65	29.0 ± 4.84	30.08 ± 5.89	7^b^
Daniel 2023	USA	Retro cohort; 3	2010 to 2020	98/102	100	100	19 (17.9–20.1)	19.9 (18.8–21)	33.4 (30.3–36.5)	48.6 (45.4–51.7)	8^b^
Darestani 2023	Iran	Retro cohort; 3	February 2015 to January 2017	160/9	90	79	30.5 ± 7.6	31.6 ± 8.3	24		7^b^
Kitchen 2022	USA	Retro cohort; 3	NR	37/43	40	40	14.9 (9.3–18.8)	15.7 (9.5–18.7)	27.6 (24-36.1)	29.0 (24-36.4)	7^b^
Mohan 2023	UK	Retro cohort; 3	NR	140/41	70	111	34.5(19–50)	30.4(18–55)	18(14–25)	20(16–25)	7^b^
Parkes 2021	USA	RCS;3	July 2011 to July 2017	75/33	36	72	25.3 ± 8.6	24.9 ± 9.6	26.1 ± 2.5	31.3 ± 12.9	7^b^
Shantanu 2019	India	Pro cohort; 2	NR	41/9	25	25	27.84 ± 9.44	32.16 ± 9.22	6		7^b^
Szakiel 2022	USA	Retro cohort; 3	January 2013 to December 2015	23/23	23	23	31.53 ± 8.37		12		7^b^
von Essen 2022	Sweden	Retro cohort; 3	NR	46/34	40	40	29.15 ± 6.83	29.15 ± 7	24		7^b^

QA, Quality Assessment (a: Modified Jadad scale; b: Newcastle–Ottawa scale); RCS, retrospective comparative study; RCT, Randomized controlled trial; Pro, prospective; Retro, retrospective; NR, not reported

**Table 2:** The graft types, suture tape products and fixation methods of the included studies

Study	Graft Type	Type of Suture tape Product	Femoral Fixation	Tibial Fixation
Allom 2022	HT(ST ± G) autograft	NR	SD	SD or IS
Aujla 2021	HT(ST + G) autograft	LARS	SD	IS
Bodendorfer 2019	HT(ST) autograft or allograft	FiberTape	SD	SD
Daniel 2023	QT, BTB, or HT autograft	FiberTape	SD	IS
Darestani 2023	HT(ST) autograft	FiberWire	SD	IS
Kitchen 2022	HT(ST + G) autograft	FiberTape	SD	IS
Mohan 2023	HT(ST + G) autograft	FiberWire, TigerWire	SD	IS
Parkes 2021	HT(ST ± G) autograft	FiberTape	SD	SD
Shantanu 2019	HT(ST + G) autograft	FiberTape	SD	NR
Szakiel 2022	HT(ST) autograft	FiberTape	SD	SD
von Essen 2022	HT(ST) or QT autograft	FiberTape	SD	SD

HT, hamstring tendon; ST, semitendinosus tendon; G, gracilis tendon; QT, quadriceps tendon; BTB, bone-patellar tendon-bone; SD, suspensory device; IS, interference screw; NR, not reported

The studies included were from six different countries: the USA, China, India, Australia, Sweden, and Iran. Most of the studies were retrospective cohort studies, with one study being prospective. A total of 8 studies [19, 30–35, 38] reported graft failure rates, and 4 studies [19, 30, 33, 35] reported RTS rates. Regarding knee function scores, 5 studies [30, 32, 33, 35, 36] provided Lysholm scores, 3 studies [30, 33, 35] reported Tegner scores, 5 studies [19, 30, 31, 34, 37] provided KOOS scores, 4 studies [19, 30, 32, 35] reported IKDC scores, 2 studies [31, 33] reported VAS scores, 2 studies [19, 33] provided SANE results, and 4 studies [29–31, 38] reported knee joint laxity.

### Quality assessment

Two independent researchers evaluated the quality of the included studies. If there were discrepancies in the summarized scores, a third researcher was responsible for resolving them. All 11 included studies were cohort studies, and their quality was assessed using NOS. The scores for all included cohort studies were greater than 7, indicating a high quality of the included literature. Detailed scoring can be found in Table 3.

**Table 3:** Quality assessment of the included studies

Author, Year	Selection	Comparability	Assessment of results	Scores
Allom 2022	4	2	2	8
Aujla 2021	3	2	2	7
Bodendorfer 2019	3	2	2	7
Daniel 2023	3	2	3	8
Darestani 2023	3	2	2	7
Kitchen 2022	3	2	2	7
Mohan 2023	3	2	2	7
Parkes 2021	3	2	2	7
Shantanu 2019	3	2	2	7
Szakiel 2022	3	2	2	7
von Essen 2022	3	2	2	7

### Clinical outcomes

#### Graft failure rate

**Fig. 2:**
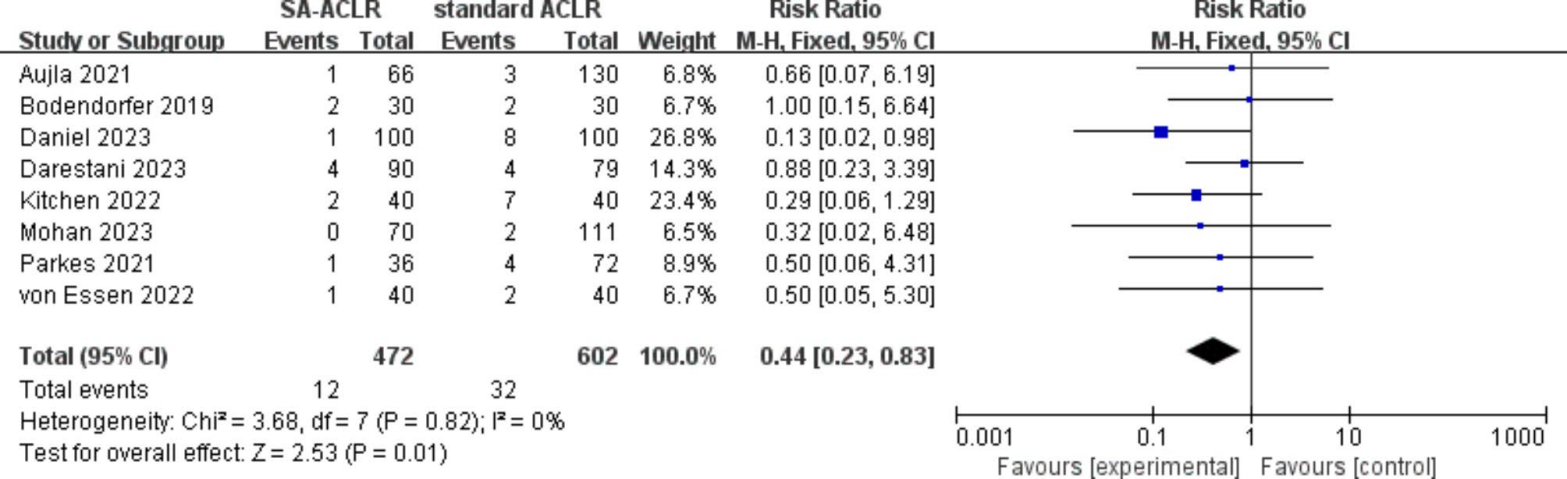
Forest plot indicates graft failure rate between ITRT + ACLR and standard ACLR group

The graft failure rate was analyzed based on the results from the latest follow-up in the included studies. A total of 8 studies [19, 30–35, 38] reported graft failure rates, encompassing 1,074 patients, with 472 in the ITRT-assisted ACLR group and 602 in the conventional ACLR group. No heterogeneity was observed among the studies (*I*² = 0%; *P* = 0.82), which justified the application of a fixed-effect model for the pooled analysis. The findings revealed that the rate of postoperative graft failure was significantly lower in the ITRT + ACLR group compared to the conventional ACLR group (OR = 0.44; 95% CI: 0.23–0.83; *P* = 0.01) (Fig. 2). It is worth noting that, due to the lack of objective imaging or second-look arthroscopic assessments of graft integrity and healing in the included studies, the result should be interpreted with caution.

#### RTS rate

**Fig. 3:**
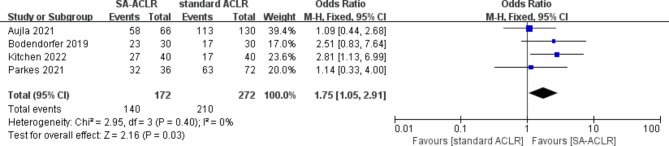
Forest plot reveals RTS rate between ITRT + ACLR and standard ACLR group

Four studies [19, 30, 33, 35] reported the RTS rate, involving a total of 444 patients (172 in the ITRT-assisted ACLR group and 272 in the conventional ACLR group). The heterogeneity test revealed no evidence of heterogeneity across the studies (*I*² = 0%; *P* = 0.40). Consequently, a fixed-effect model was employed for the pooled analysis, which demonstrated that the ITRT + ACLR group achieved a superior postoperative RTS rate compared to the conventional ACLR group (OR = 1.75; 95% CI: 1.05–2.91; *P* = 0.03) (Fig. 3).

#### Tegner activity score

**Fig. 4:**
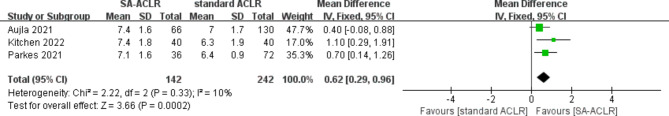
Forest plot indicates Tegner score between ITRT + ACLR and standard ACLR group

Three studies [30, 33, 35] reported Tegner score results, involving a total of 384 patients (142 in the ITRT-assisted ACLR group and 242 in the conventional ACLR group). The heterogeneity test indicated low heterogeneity among the studies (*I*² = 10%; *P* = 0.33), allowing for the use of a fixed-effect model. The analysis revealed that the postoperative Tegner scores were significantly higher in the ITRT + ACLR group compared to the conventional ACLR group (MD = 0.62; 95% CI: 0.29–0.96; *P* = 0.002) (Fig. 4).

#### KOOS score

**Fig. 5:**
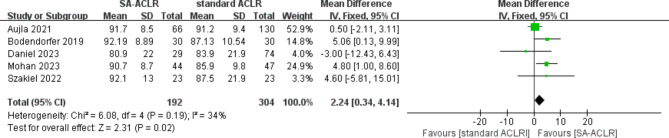
Forest plot illustrates KOOS score between ITRT + ACLR and standard ACLR group

The KOOS scores were analyzed based on the latest follow-up results from five studies [19, 30, 31, 34, 37], involving a total of 496 patients (192 in the ITRT-assisted ACLR group and 304 in the conventional ACLR group). The heterogeneity test indicated low heterogeneity among the studies (*I*² = 34%; *P* = 0.19), allowing the use of a fixed-effect model. The analysis results demonstrated that the ITRT-assisted ACLR group had significantly better postoperative KOOS scores compared to the conventional ACLR group (MD = 2.24; 95% CI: 0.34–4.14; *P* = 0.02) (Fig. 5).

#### Lysholm score

**Fig. 6:**
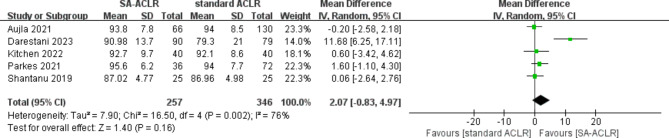
Forest plot indicates Lysholm score between ITRT + ACLR and standard ACLR group

Five studies [30, 32, 33, 35, 36] reported Lysholm score results, involving a total of 603 patients (257 in the ITRT-assisted ACLR group and 346 in the conventional ACLR group). Given the significant heterogeneity observed among the studies (*I*² = 76%; *P* = 0.002), a random-effects model was applied for the pooled analysis. The findings showed no significant difference in postoperative Lysholm scores between the ITRT-assisted ACLR group and the conventional ACLR group (MD = 2.07; 95% CI: -0.83-4.97; *P* = 0.16) (Fig. 6). Furthermore, we found that after excluding individual studies, the pooled results showed no significant changes, indicating that the meta-analysis results for the Lysholm score were stable.

#### IKDC score

**Fig. 7:**
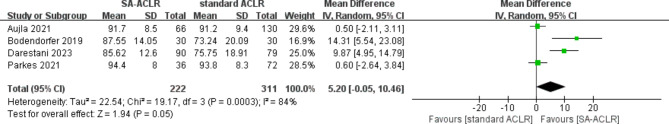
Forest plot indicates IKDC score between ITRT + ACLR and standard ACLR group

Four studies [19, 30, 32, 35] reported IKDC score results, involving a total of 533 patients (222 in the ITRT-assisted ACLR group and 311 in the conventional ACLR group). Due to significant heterogeneity among the studies (*I*² = 84%; *P* = 0.0003), a random-effects model was used for the analysis. The results indicated no significant difference in IKDC scores between the ITRT + ACLR group and the conventional ACLR group (MD = 5.20; 95% CI: -0.05-10.46; *P* = 0.05) (Fig. 7). Moreover, we found that after excluding individual studies, the pooled results showed no significant changes, indicating that the meta-analysis results for the IKDC score were stable.

#### VAS score

**Fig. 8:**
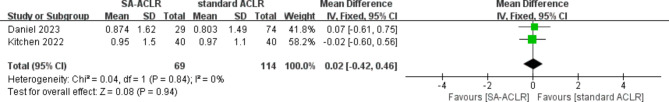
Forest plot shows VAS score between ITRT + ACLR and standard ACLR group

Two studies [31, 33] provided VAS score data at the final follow-up, encompassing a total of 183 patients, with 69 in the ITRT-assisted ACLR group and 114 in the conventional ACLR group. The heterogeneity test showed no evidence of heterogeneity between the studies (*I*² = 0%; *P* = 0.84), justifying the use of a fixed-effect model. The analysis indicated no significant difference in postoperative VAS scores between the two surgical approaches (MD = 0.02; 95% CI: -0.42-0.46; *P* = 0.94) (Fig. 8).

#### SANE score

**Fig. 9:**
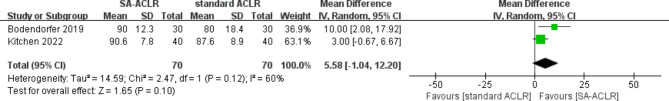
Forest plot indicates SANE score between ITRT + ACLR and standard ACLR group

Two studies [19, 33] reported SANE score results, involving a total of 140 patients (70 in the ITRT-assisted ACLR group and 70 in the conventional ACLR group). The heterogeneity test indicated moderate heterogeneity between the studies (*I*² = 60%; *P* = 0.12), so a random-effects model was used for the pooled analysis. The analysis results showed no significant difference in postoperative SANE scores between the two treatment methods (MD = 5.58; 95% CI: -1.04-12.20; *P* = 0.10) (Fig. 9).

#### Knee joint laxity

**Fig. 10:**
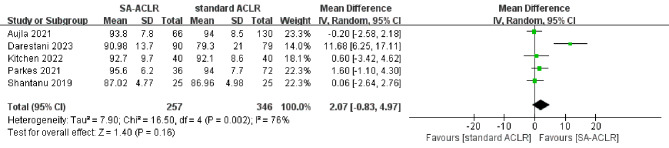
Forest plot indicates SSD between ITRT + ACLR and standard ACLR group

Knee joint laxity was reported in four studies [29–31, 38], which measured the anterior-posterior laxity difference between the operated and contralateral limbs using the KT-1000/2000 device, known as the side-to-side difference (SSD). In total, 407 patients were assessed, with 214 in the ITRT-assisted ACLR group and 193 in the conventional ACLR group. The heterogeneity test revealed moderate heterogeneity across the studies (I² = 58%; *P* = 0.07), prompting the use of a random-effects model for the pooled analysis. The findings indicated no statistically significant difference in postoperative knee joint laxity between the ITRT-assisted ACLR and conventional ACLR groups (MD = 0.00; 95% CI: -0.31-0.31; *P* = 1.00) (Fig. 10).

## Discussion

The most significant finding of this study is that ITRT-assisted ACLR, compared to conventional ACLR, is associated with a lower graft failure rate and improved knee function outcomes, including a higher return-to-sport (RTS) rate, better KOOS scores, and higher Tegner activity scores (Table 4). However, no studies specifically reported whether ITRT-assisted ACLR reduces graft load and the risk of rupture, which prevented further analysis of this potential correlation. Overall, patients undergoing ITRT-assisted ACLR achieved favorable clinical outcomes and functional results.

**Table 4:** The meta-analysis results were summarized

Clinical outcome measures	Number of studies	Patients (ITRT + ACLR vs. ACLR)	OR/MD	95% CI	*P* value
Graft failure rate	8	472 vs. 602	0.44	0.23–0.83	0.01
RTS rate	4	172 vs. 272	1.75	1.05–2.91	0.03
Tegner activity score	3	142 vs. 242	0.62	0.29–0.96	0.002
KOOS score	5	192 vs. 304	2.24	0.34–4.14	0.02
Lysholm score	5	257 vs. 346	2.07	-0.83-4.97	0.16
IKDC score	4	222 vs. 311	5.2	-0.05-10.46	0.05
VAS score	2	69 vs. 114	0.02	-0.42-0.46	0.94
SANE score	2	70 vs. 70	5.58	-1.04-12.20	0.1
Knee joint laxity	4	214 vs. 193	0	-0.31-0.31	1

In recent years, using tension-relieving devices to enhance the biomechanical properties of ACL grafts has become a popular topic in sports medicine [39]. During the early stages of postoperative rehabilitation, the graft becomes “fragile” due to processes of necrosis and remodeling, and the use of ITRT aims to distribute excessive loads on graft [40]. Bachmaier et al. [22] found that adding a tension-relieving band improved the mechanical performance of ACL grafts by sharing additional loads without negatively affecting bone tunnel healing. However, independent reinforcement in ACLR reduces graft elongation and increases the failure load, with this effect being more pronounced and occurring earlier in smaller-diameter grafts. A cadaveric study further indicated that, compared to conventional ACLR, ACLR with a tension-relieving device enables full-range motion, improves graft stiffness, and enhances knee joint stability [23]. One of the primary purposes of using ITRT is to protect the graft and reduce the risk of graft failure. In this study, the graft failure rate in the ITRT-assisted ACLR group was lower than that in the conventional reconstruction group. Daniel et al. [31] conducted a retrospective study involving 100 patients with ITRT-assisted ACLR and 100 patients with conventional ACLR, with a follow-up period of at least 24 months. They found that although the knee functional outcomes were similar between the two groups, ITRT-assisted ACLR reduced the risk of revision after ACLR. Kitchen et al. [33] followed up with 80 adolescent patients who underwent ACLR (40 in the ITRT-assisted ACLR group and 40 in the conventional ACLR group) for at least 2 years. The study showed that the ITRT-assisted ACLR group had higher subjective functional scores and a lower early failure rate, with a significantly better Tegner score compared to the conventional group (*P* = 0.017).

Several previous meta-analyses have reported the use of ITRT in ACLR, showing beneficial clinical outcomes. Vermeijden et al. [41] found that internal tension-reducing enhanced ACL repair is a reliable option for treating ACL tears, with favorable knee functional outcomes in short-term follow-up. Regarding functional outcomes in both patient groups, our study showed that almost all subjective scores improved to some extent at the final follow-up, indicating an improvement in clinical symptoms. The ITRT-assisted ACLR group had an advantage in KOOS scores and Tegner scores, suggesting that patients using the internal tension-reducing device could better maintain their level of physical activity. Four studies included in our analysis [29–31, 38] reported improvements in objective knee stability. However, contrary to expectations, the meta-analysis of these studies revealed that the use of the internal tension-reducing device did not show any advantage in objective stability compared to conventional ACLR. Therefore, while adding a tension-reducing device to the graft may enhance its biomechanical properties, it does not necessarily lead to better objective knee stability.

In terms of the RTS rate, a recent systematic review that included nine studies involving 314 patients found that patients who underwent suture tape-enhanced ACL reconstruction had a higher return-to-sport rate compared to those with isolated ACL reconstruction, but there was no significant difference in graft failure rates between the two groups [42]. Similarly, we found that ITRT-assisted ACLR showed superiority over conventional ACLR in terms of the return-to-sport rate. A possible explanation for the better postoperative athletic performance in ITRT-assisted ACLR patients is that their awareness of using an internal tension-reducing device might give them greater confidence in returning to sports [43]. Moreover, there were no statistically significant differences between the two groups in VAS scores, SANE scores, Lysholm scores, or IKDC scores, indicating that there was no difference in postoperative pain levels or certain functional outcomes regardless of the use of ITRT. A recent systematic review also concluded that the current evidence is insufficient to support the notion that ITRT-assisted ACLR leads to superior clinical outcomes compared to isolated ACLR [44]. Similarly, Mackenzie et al. [24] included six biomechanical studies, three animal studies, ten technical studies, and three clinical studies, finding that the postoperative clinical functional outcomes of ITRT-assisted ACLR patients were similar to those of conventional ACLR patients. Additionally, Bodendorfer et al. [19] reported no significant differences in graft failure rates or complication rates between the two groups at the final follow-up. Parkes et al. [35] reached a consistent conclusion, further pointing out that a total sample size of 1,290 patients, including at least 430 in the ITRT-assisted ACLR group, would be required to detect differences in graft failure rates between the two groups. Therefore, higher-quality evidence, larger sample sizes, and longer follow-up results are still needed to support the superiority of ITRT-assisted ACLR in clinical outcomes and knee function.

Additionally, a crucial consideration in ACL reconstruction is its long-term impact on joint health, particularly the development of post-traumatic osteoarthritis (PTOA). ACL injuries are known to increase the risk of PTOA, even after reconstruction [45]. ACL biomechanical studies suggested that improved load distribution and enhanced graft stiffness, as facilitated by ITRT, might mitigate the risk of chronic joint instability, a factor associated with PTOA progression [46–48]. However, evidence linking ITRT-assisted ACLR to a reduced incidence of PTOA remains scarce. Future studies with extended follow-up periods are necessary to evaluate whether the biomechanical advantages of ITRT translate into meaningful reductions in the prevalence or severity of PTOA. Understanding this long-term benefit would be critical for assessing the overall utility of ITRT in clinical practice.

This study has several definite limitations: (1) The lack of high-level clinical studies, with most included studies being of low evidence level (Grade III) and the risk of bias inherent in retrospective studies, reduces the validity of this research. To address this limitation, larger-scale multicenter randomized controlled trials will be needed in the future; (2) None of the included studies utilized imaging examinations or second-look arthroscopy to assess intra-articular conditions after ITRT-assisted ACLR; (3) The follow-up period in all included studies was relatively short, with a lack of medium- and long-term follow-up reports. Additionally, some clinical outcomes were reported by only one or two studies, making it impossible to perform a pooled analysis.

## Conclusions

This systematic review and meta-analysis showed that ITRT-assisted ACLR has significant advantages over conventional ACLR in reducing graft failure rates and improving knee function. Future randomized controlled trials with longer follow-up durations are needed to further investigate the long-term efficacy and functional outcomes of ITRT-assisted ACLR.

## Data Availability

No datasets were generated or analysed during the current study.
